# An important discovery on combination of irreversible electroporation and allogeneic natural killer cell immunotherapy for unresectable pancreatic cancer

**DOI:** 10.18632/oncotarget.21974

**Published:** 2017-10-19

**Authors:** Mao Lin, Mohammed Alnaggar, Shuzhen Liang, Xiaohua Wang, Yinqing Liang, Mingjie Zhang, Jibing Chen, Lizhi Niu, Kecheng Xu

**Affiliations:** ^1^ Fuda Cancer Hospital, School of Medicine, Jinan University, Guangzhou, China; ^2^ Fuda Cancer Institute, Guangzhou, China; ^3^ Hank Bioengineering Co., Ltd, Shenzhen, China

**Keywords:** clinical efficacy, irreversible electroporation, allogeneic natural killer cell, pancreatic cancer

## Abstract

**Purpose:**

To study the safety and clinical efficacy on combination of irreversible electroporation and allogeneic natural killer cell therapy for treating Stage III/IV pancreatic cancer, evaluating median progression free survival (PFS), and overall survival (OS).

**Results:**

Adverse events of all patients were limited to grades 1 and 2, including local (mainly tussis 13.4%, nausea and emesis 7.1%, pain of puncture point 29.6% and duodenum and gastric retention 4.3%) and systemic (mainly fatigue 22.3%, fever 31.6%, and transient reduction of intraoperative blood pressure 25.1% and white cell count reduction 18.3%) reactions, fever was the most frequent. The serum amylase level at 24 h and 7 d after IRE was not significantly changed compared to those before IRE (*P* > 0.05). CA19–9 value was lower in IRE-NK group than in IRE at 1 month after treatment (*P* < 0.05). After a median follow-up of 7.4 months (3.6–11.2 months): in stage III group, median PFS was higher in IRE-NK group (9.3 months) than in IRE group (8.1 months, *P* = 0.0465), median OS was higher in IRE-NK (13.2 months) than in IRE (11.4 months, *P* = 0.0411), and median PFS was higher in who received multiple NK than single NK (9.8 months vs.8.1 months, *P* = 0.0423, respectively), median OS who received multiple NK was higher than single NK (13.9 months vs.12.3 months, *P* = 0.0524, respectively), the RR in IRE-NK (63.2%) was higher than in IRE (50.0%, *P* < 0.05); in stage IV group, median OS was higher in IRE-NK (9.8 months) than in IRE (8.7 months, *P* = 0.0397), the DCR in IRE-NK (66.7%) was higher than in IRE (42.9%, *P* < 0.05).

**Materials and Methods:**

Between July 2016 and May 2017, we enrolled 71 patients who met the enrollment criteria. The patients were divided into stage III (32 patients, 17 patients received only IRE and 15 patients received IRE-NK (Irreversible electroporation- natural killer): 8 patients underwent a course of NK and 7 patients underwent ≥ 3 courses) and stage IV (39 patients, 22 patients received only IRE and 17 patients received IRE-NK: 9 patients underwent a course of NK and 8 patients underwent ≥ 3 courses). The safety and short-term effects were evaluated firstly, then the median PFS, median OS, response rate (RR) and disease control rate (DCR) were assessed.

**Conclusions:**

Combination of irreversible electroporation and allogeneic natural killer cell immunotherapy significantly increased median PFS and median OS in stage III pancreatic cancer and extended the median OS of stage IV pancreatic cancer. Multiple allogeneic natural killer cells infusion was associated with better prognosis to stage III pancreatic cancer.

## INTRODUCTION

Pancreatic cancer (PC) is one of the most malignant cancers which usually discovered late and has a poor prognosis [1]. Although the resection rate of pancreatic cancer has improved in recent years, postoperative pancreatic fistula, bleeding and other complications are still more, and often because tumor invasion, surrounding blood vessels bile duct resection and other important pipeline structure due to limited resection range. Therefore, systemic palliative chemotherapy (such as gemcitabine) is often used, however, the sensitivity of pancreatic cancer to chemotherapy is poor, and the prognosis is still poor [2–4]. Approximately 40% of patients present with locally advanced pancreatic cancer (LAPC), the estimated rates of 1- and 5-year survival of LAPC are 24% and 4.3%, respectively [5]. Therefore, identifying more effective therapies for patients with unresectable PC remains an important clinical challenge.

Aimed at unresectable PC, the traditional ablation method such as cryoablation, which has been used and showed a good effect assuredly but also damaged the peripheral blood vessels, bile ducts and pancreatic ducts and other important structures easily [6, 7]. The incidence of postoperative hemorrhage and pancreatic fistula was high, and peripheral tumor ablation is not complete [8]. However, Irreversible electroporation (IRE) is an emerging, non-thermal, image-guided tumor ablation technique that has been proven feasible and safe for treating locally advanced pancreatic tumors [9–12]. IRE is a newly developed method that causes apoptosis without injuring the structural components of tissues. Fuda Cancer Hospital (Guangzhou, China) has been conducting a prospective study of percutaneous IRE ablation of advanced pancreatic cancer (APC) earliest since approved by CFDA. However, as the vast majority of patients with APC patients have distal metastasis, and IRE technology can only treat primary tumors, tumor cells can reach any tissue or organ via the hematogenous spread.

Some previous reports have demonstrated that cancer development and progression in APC patients are associated with the tumor immune [13–18]. Immunotherapy benefit in APC patients has been studied for many years [19–21]. However, due to the expression of MHC down-regulated, tumor cells often appeared immune escape [22]. As we all known, NK cells play an important role against foreign matter earliest including cancer [23, 24]. With understanding NK function, NK cell transfer has promised anti-tumor effects on various tumors [25–29], including pancreatic cancer [30–34].

In this study, we prospectively investigated the clinical response of IRE combined with NK cell immunotherapy in patients with unresectable (Stage III/ IV) pancreatic cancer to provide a potential therapeutic pattern.

## RESULTS

### Patient demographics and related data during IRE

Pre-treatment information was collected from the 71 patients, who were from China (*n* = 35), Indonesia (*n* = 7), Malaysia (*n* = 14), the Middle East (*n* = 9) and other countries (*n* = 6), with a median age of 57.0 years (range, 41–73 years). Among them, adenocarcinoma was detected in 46 patients. 49 of the 71 patients had the previously received chemotherapy (Gemcitabine, *n* = 34; FOLFIRINOX, *n* = 15), with a median of four cycles (range, 2 to 6 cycles). Patients underwent a 105.1 ± 32.4 (39~202) minute surgical procedure and 9.3 ± 4.9 (3~21) ablation cycle. According to the seventh edition of the American Joint Committee on Cancer (AJCC), the data of the two groups were compared, and the patient demographics were not statistically different (Table 1). The related operative data of the patients receiving IRE showed that anesthesia was smooth, the electrocardiogram (ECG) monitor displayed myocardial contractions within the absolute refractory period, and the patient vital signs were stable (Table 2).

**Table 1:** Patient demographics

Patient characteristics pre-treatment	Stage III (*n* = 32)	Stage IV (*n* = 39)	*P* value
**Sex (male/female)**	17/15	19/20	*P* = 0.764
**Median age (y)**	55	59	*P* = 0.788
**Pathology**			*P* = 0.869
Adenocarcinoma	23	26	
**Tumor location**			*P* = 0.911
Head and neck	23	26	
Body and tail	6	5	
**Tumor size before IRE**	5.01 ± 1.06	4.92 ± 1.38	*P* = 0.864
**Karnofsky performance status**			*P* = 0.587
70	13	19	
80	14	16	
90	5	4	
**Chemotherapy**	29	37	*P* = 0.564

**Table 2:** Related operative data of patients receiving IRE (*n* = 67, mean ± standard deviation)

Intraoperative data	X ± S	Range
**Ablation duration (min)**	105.1 ± 32.4	39.0–202.0
**Perioperative bleeding (mL)**	152.0 ± 45.0	90.0–230.0
**Electrode needle spacing (cm)**	1.8 ± 0.3	1.5–2.5
**Ablation times /(time)**	9.3 ± 4.9	3.0–23.0
**Average voltage (V)**	2635.0 ± 165.2	2400.0–3000.0
**Average pulse width (μs)**	78.9 ± 6.4	70.0–90.0

### Safety evaluation

Throughout the trial, adverse events of all patients were limited to grades 1 and 2, included local (mainly tussis 13.4%, nausea and emesis 7.1%, pain of puncture point 29.6% and duodenum and gastric retention 4.3%) and systemic (mainly fatigue 22.3%, fever 31.6%, and transient reduction of intraoperative blood pressure 25.1% and white cell count reduction 18.3%) reactions, fever was the most frequent (Table 3). Symptomatic treatment relieved all symptoms within the day and the symptoms did not reappear. There was no pancreatic fistula, bleeding, bile leakage, or abdominal cavity infection at 2 months post-treatment.

**Table 3:** Adverse events for all patients

Adverse events	Case (*n*)	Percentage (%)	Grade
**Local reactions**			
Tussis	10	13.4	1
Nausea and emesis	5	7.1	1
Pain of puncture point	21	29.6	1
Duodenum and gastric retention	3	4.3	2
**Systemic reactions**			
Fatigue	16	22.3	1
Fever	22	31.6	1
Transient reduction of intraoperative blood pressure	18	25.1	2
White cell count reduction	13	18.3	2

**Figure 1:**
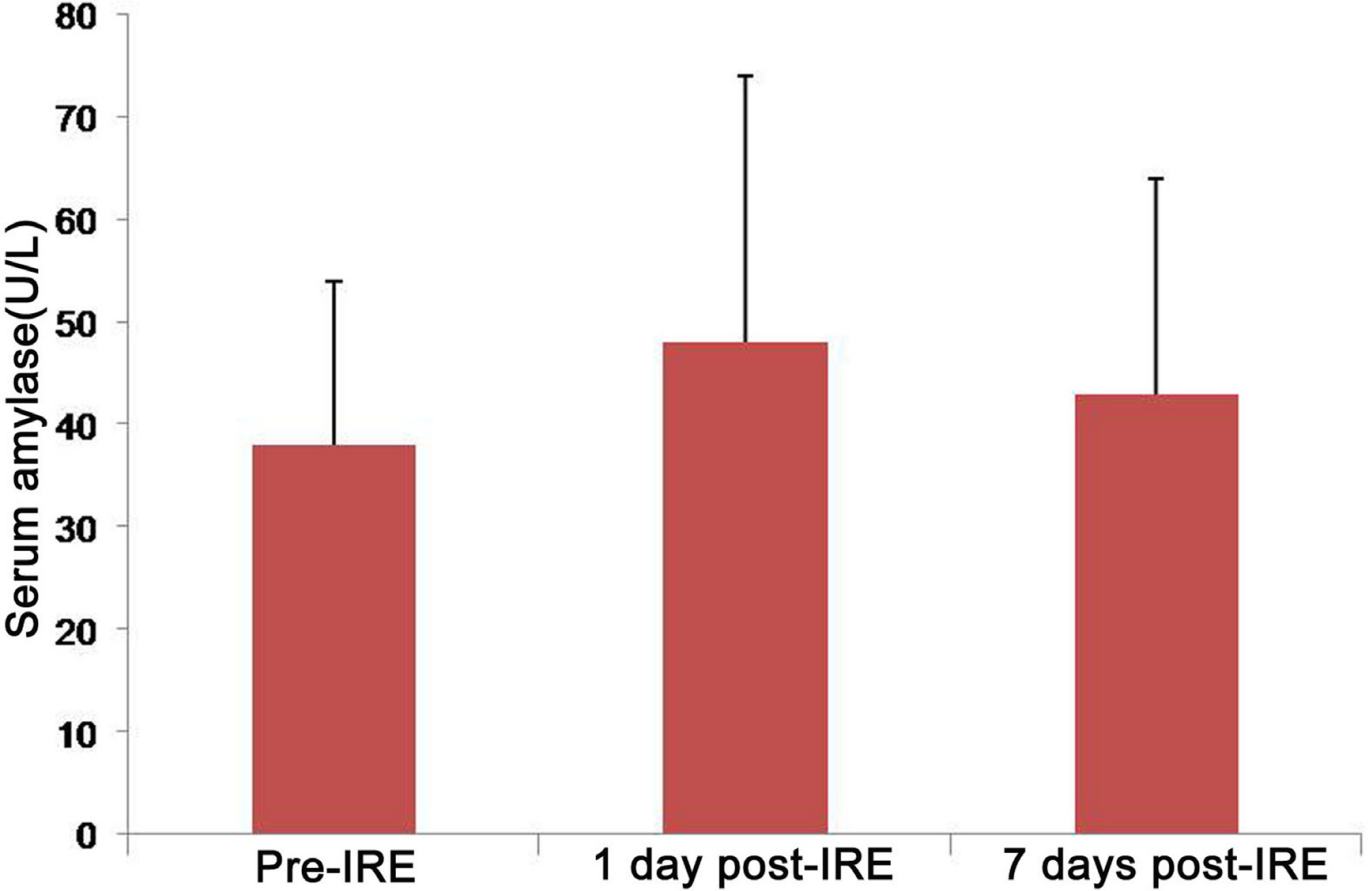
Error bar chart shows amylase values before and after IRE Compared with pre-IRE, there was no significant increase in amylase levels at 1 day after IRE and 7 day after IRE (*P* > 0.05).

The serum amylase levels of all patients at 1 day pre-treatment were normal (0~220 U/L). Compared with pre-IRE, there was no significant increase in amylase levels at 1 day after IRE and 7 day after IRE (*P* > 0.05, Figure 1).

### Clinical efficacy

The pre-treatment immune test data of were merged and compared with post-treatment (Table 4). For lymphocyte count, all subsets were significantly higher in the IRE-NK group post-treatment, especially NK (*P* < 0.001); for lymphocyte function, Th1 cytokine levels were higher in the IRE-NK group, while Th2 cytokine levels were essentially unchanged.

**Table 4:** Comparison of lymphocyte number and function

	Stage III				Stage IV		
Lymphocyte test items	Pre-IRE (*n* = 35)	IRE (*n* = 16)	IRE-NK (*n* = 19)	Lymphocyte test items	Pre-IRE (*n* = 32)	IRE (*n* = 14)	IRE-NK (*n* = 18)
**Number (cell/μL)**				**Number (cell/μL)**			
**Total T cell**	1329 ± 54	1578 ± 69^*^	1900 ± 72^**^	**Total T cell**	1258 ± 49	1287 ± 57^*^	1456 ± 94^*^
**CD8 + T cell**	614 ± 12	709 ± 37^*^	778 ± 15^*^	**CD8 + T cell**	614 ± 11	632 ± 26	745 ± 15^*^
**CD4 + T cell**	726 ± 31	808 ± 35^*^	861 ± 33^**^	**CD4 + T cell**	735 ± 30	766 ± 35	802 ± 36
**NK cell**	409 ± 38	456 ± 61	658 ± 73^***^	**NK cell**	389 ± 37	392 ± 46	569 ± 69^***^
**B cell**	309 ± 11	465 ± 33^**^	551 ± 41^*^	**B cell**	334 ± 10	365 ± 33	351 ± 38
**Function (pg/mL)**				**Function (pg/mL)**			
**IL-2**	9.1 ± 3.3	16.9 ± 3.9^**^	23.1 ± 4.6^***^	**IL-2**	9.9 ± 3.1	10.6 ± 3.5	17.1 ± 4.8^**^
**TNF-β**	3.6 ± 2.4	9.4 ± 2.1^***^	14.6 ± 2.8^***^	**TNF-β**	3.8 ± 2.1	9.8 ± 2.2	13.7 ± 2.6^**^
**IFN-γ**	3.9 ± 3.1	11.1 ± 2.6^**^	15.2 ± 3.8^***^	**IFN-γ**	4.8 ± 2.8	10.9 ± 3.1	13.1 ± 29^*^
**IL-4**	10.2 ± 2.1	10.1 ± 3.4	11.2 ± 3.1	**IL-4**	9.7 ± 2.3	10.5 ± 2.4	10.2 ± 3.7
**IL-6**	13.3 ± 3.9	15.1 ± 4.5	15.5 ± 5.9^**^	**IL-6**	11.2 ± 3.2	12.2 ± 3.7	12.6 ± 3.9
**IL-10**	9.4 ± 2.7	9.7 ± 2.3	10.3 ± 3.2	**IL-10**	8.9 ± 3.1	9.3 ± 2.6	12.1 ± 3.1

Every cell subset or cytokine was analyzed by Dunnett's multiple comparison test (one-way analysis of variance). NK cell, natural killer cell; IL, interleukin; TNF, tumor necrosis factor; IFN, interferon; ^*^P < 0.05; ^**^P < 0.01; ^***^P < 0.001.

**Figure 2:**
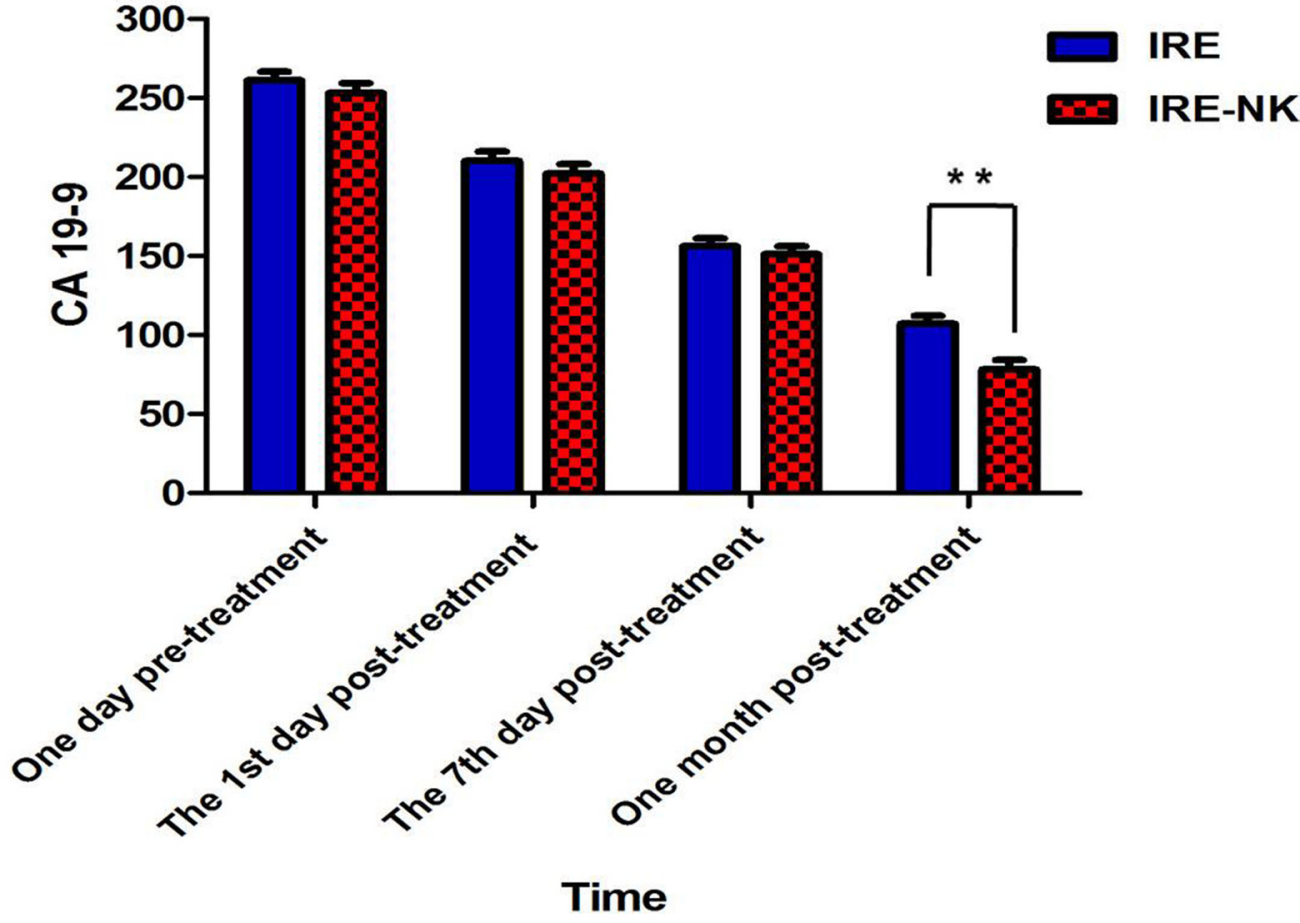
Change of CA 19–9 There was no difference between IRE and IRE-NK group at day 1, and 7 post-treatment (*P* > 0.05), but at 1 month post-treatment, CA19–9 expression was lower in the IRE-NK than in the IRE (*P* < 0.05).

CA19–9 value of all patients were higher than normal at 1 day pre-treatment and decreased gradually at day 1, 7, and 1 month post-treatment in the two groups, but still higher than normal (Figure 2). There was no difference between IRE and IRE-NK group at day 1, and 7 post-treatment (*P* > 0.05), but at 1 month post-treatment, CA19–9 expression was lower in the IRE-NK than in the IRE (*P* < 0.05).

**Figure 3:**
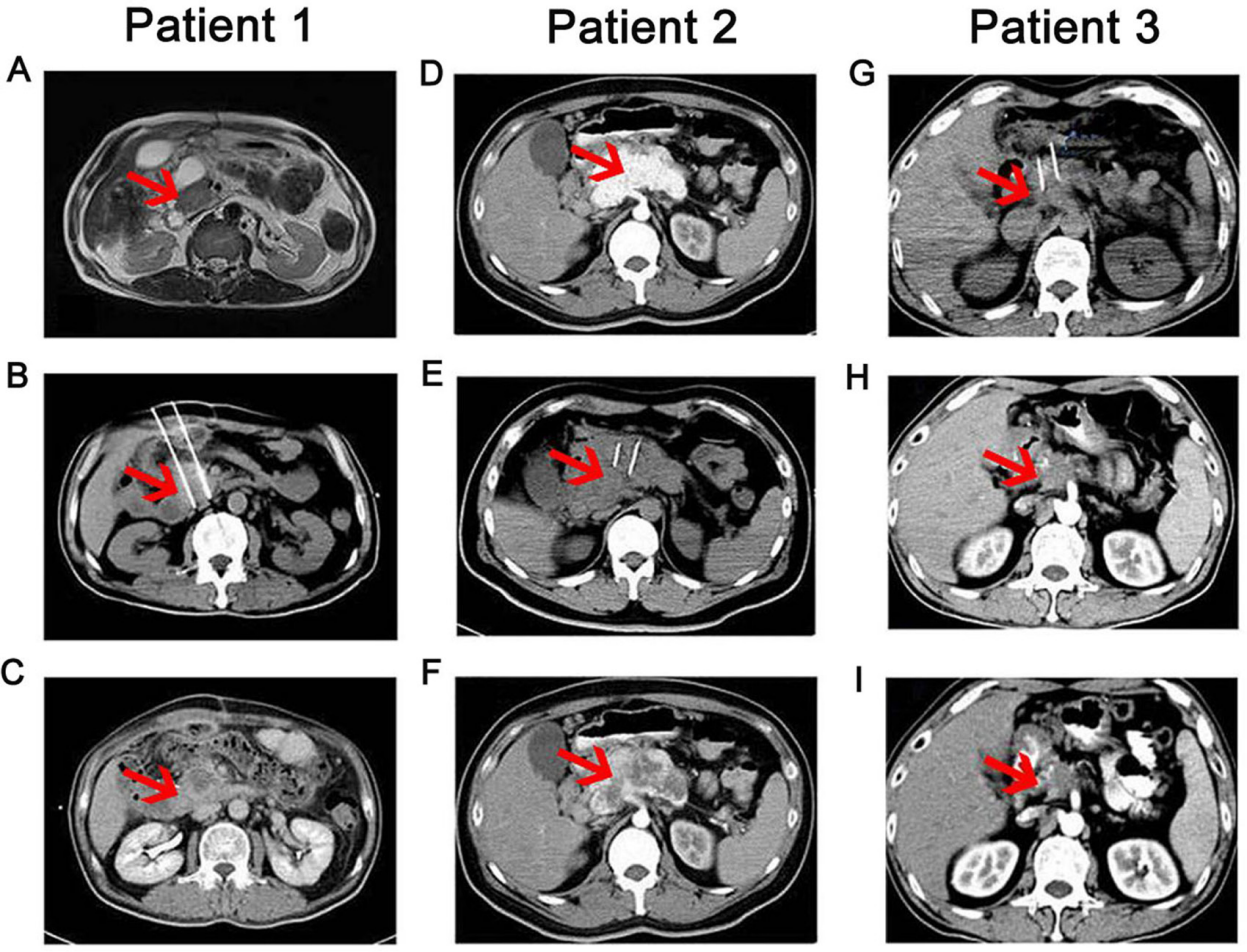
CT scans taken before, during and after IRE-NK therapy Patient 1: (**A**–**C**) (Female patient, 70 years old, T4N1M0, stage III): A. Pre-IRE CT showed a compressed duodenum and encased common bile duct and pancreatic duct due to the tumor, which was 3.1 × 2.2 cm (red arrow) in dimension; B. during IRE, two electrodes were inserted and the distance between them was 2.0 cm, as shown in this CT image; C. three month post-IRE-NK, CT showed no enhancement in the occupying lesion, with mild shrinkage of the area. Patient 2: (**D**–**F**) (Male patient, 59 years old, T4N1M1, stage IV): D. A contrast-enhanced CT scan taken before IRE showed a 5.7 × 4.2 cm contrast-enhanced lesion (red arrow) in the neck and body of the pancreas; (E) Two IRE electrodes were inserted into the tumor; F. CT scan taken two months after IRE-NK showed a 5.7 × 4.0 cm lesion with a large area of necrosis in the neck and body of the pancreas. Patient 3: (**G-I)** (male patient, 51 years old, T4NxM0, stage III): G. pancreatic head carcinoma, tumor size was about 4.1 × 3.5 cm (red arrow) and performed IRE ablation; H. 2 months after IRE, the tumor was reduced to 3.3 × 2.4 cm; I. 3 months after IRE, the tumor was reduced to 2.4 × 1.8 cm.

The clinical response was observed at 2 months post-treatment (Table 5). In stage III group, the RR in IRE-NK (66.7%) was higher than in IRE (47.1%, *P* < 0.05), but no difference on DCR (*P* > 0.05); In stage IV, the DCR in IRE-NK (70.6%) was higher than in IRE (59.1%, *P* < 0.05), but no difference on RR (*P* > 0.05). Representative results from two patients who received IRE-NK therapy were shown in Figure 3.

**Table 5:** Clinical response

	Total	Stage III		*P*-value	Total	Stage IV		*P*-value
		IRE	IRE-NK			IRE	IRE-NK	
Number	32	17	15	*P* > 0.05	39	22	17	*P* > 0.05
CR	7	3	4	*P* > 0.05	0	0	0	*P* > 0.05
PR	11	5	6	*P* > 0.05	13	8	5	*P* > 0.05
SD	7	5	2	*P* > 0.05	12	5	7	*P* > 0.05
PD	7	4	3	*P* > 0.05	14	9	5	*P* > 0.05
RR (%)	56.3	47.1	66.7	*P* < 0.05	33.3	36.4	29.4	*P* > 0.05
DCR (%)	78.1	76.5	80.0	*P* > 0.05	64.1	59.1	70.6	*P* < 0.05

Clinical responses were evaluated according to Response Evaluation Criteria in Solid Tumors version 1.1. CR, complete response; PR, partial response; SD, stable disease; PD, progressive disease; RR, response rate; DCR, disease control rate.

### Follow-up

**Figure 4:**
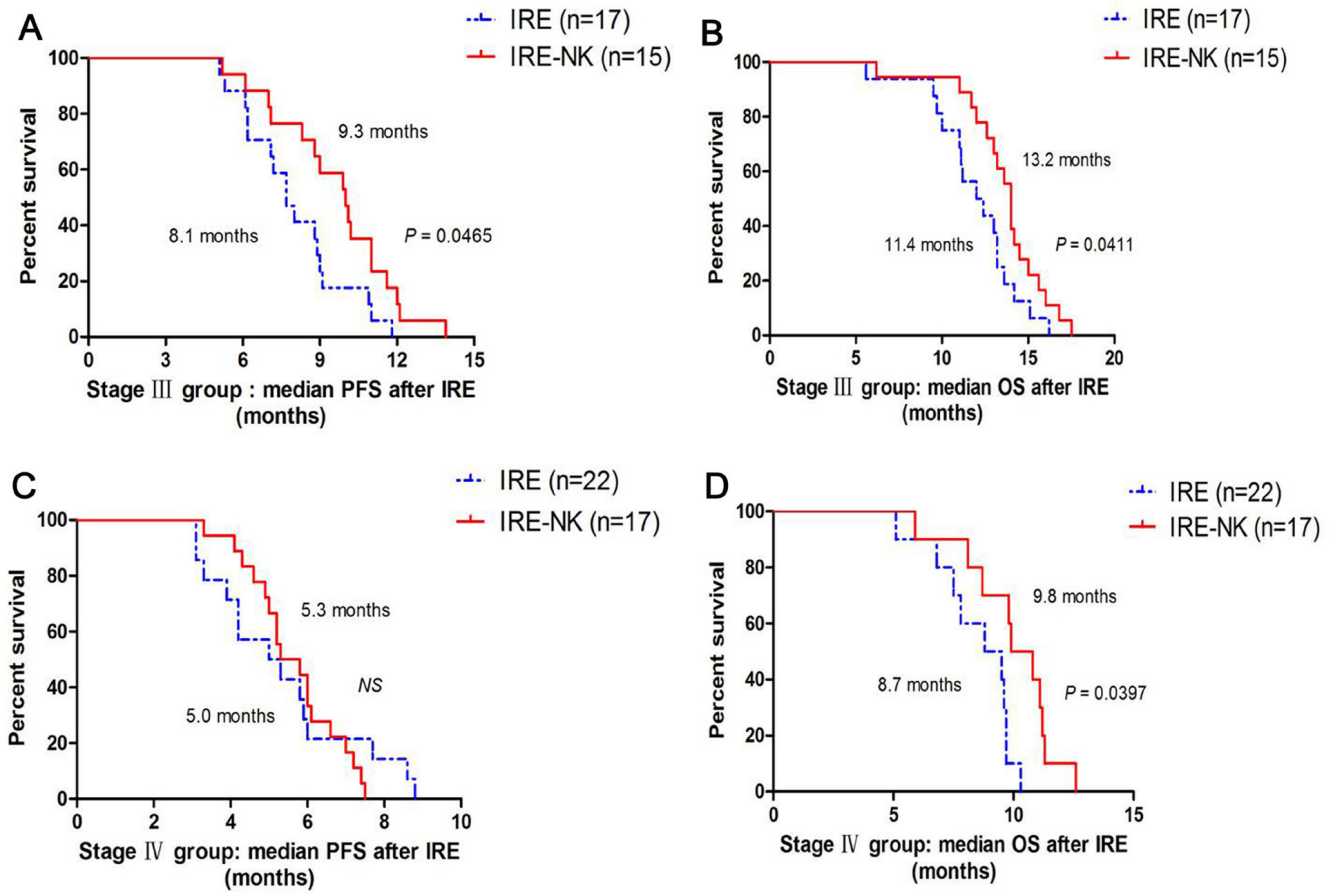
Correlation of median PFS and OS with type of treatment (**A**) Comparison of median PFS between 17 patients who underwent IRE and 15 patients who underwent IRE-NK in stage III group; (**B**) Comparison of median OS between 17 patients who underwent IRE and 15 patients who underwent IRE-NK in stage III group; (**C**) Comparison of median PFS between 22 patients who underwent IRE and 17 patients who underwent IRE-NK in stage IV group; (**D**) Comparison of median OS between 22 patients who underwent IRE and 17 patients who underwent IRE-NK in stage IV group.

**Figure 5:**
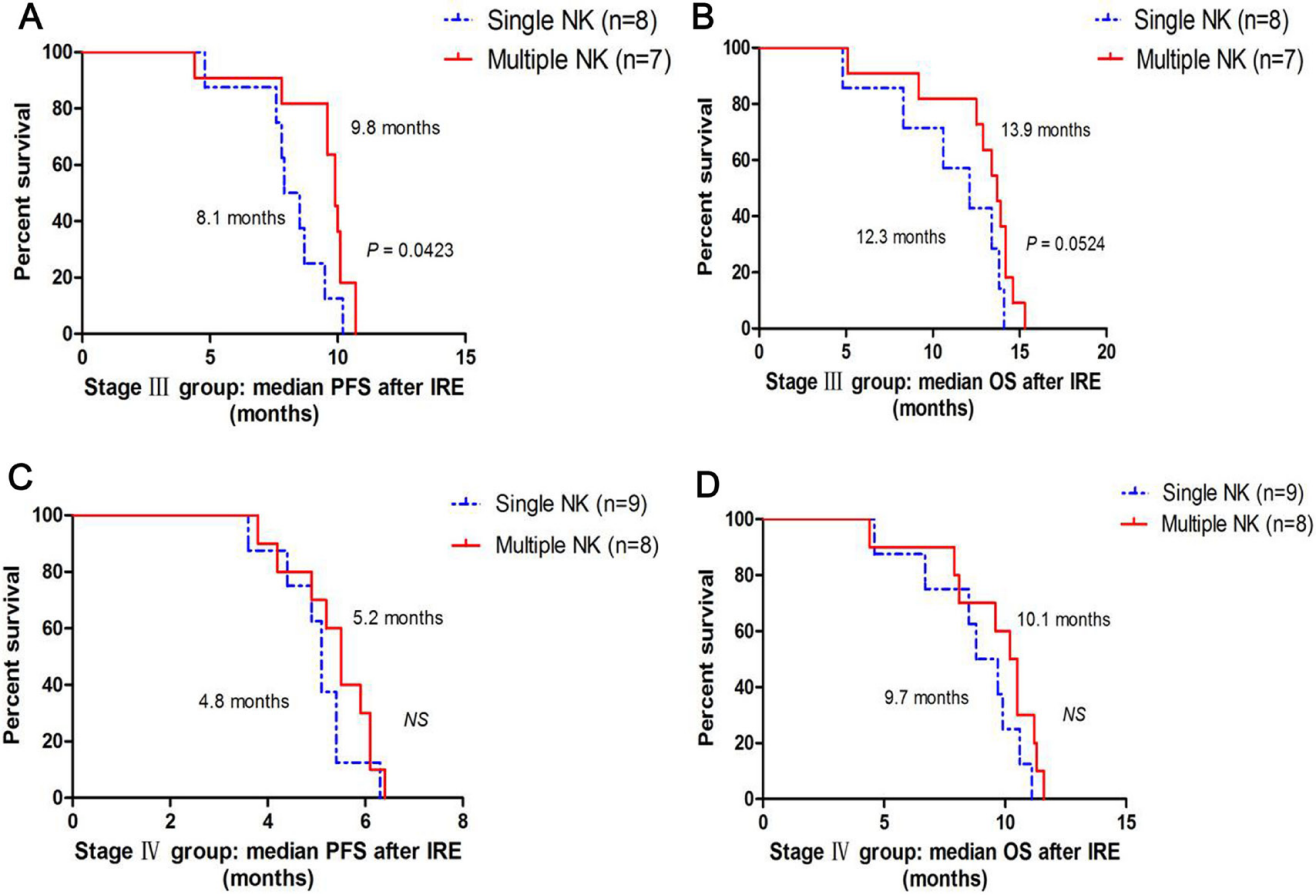
Correlation between median PFS and OS with courses of NK (**A**) Comparison of median PFS between 8 patients who underwent single NK and 7 patients who underwent multiple NK in stage III group; (**B**) Comparison of median OS between 8 patients who underwent single NK and 7 patients who underwent multiple NK in stage III group; (**C**) Comparison of median PFS between 9 patients who underwent single NK and 8 patients who underwent multiple NK in stage IV group; (**D**) Comparison of median OS between 9 patients who underwent single NK and 8 patients who underwent multiple NK in stage IV group.

After a median follow-up of 7.4 months (3.6 –11.2 months): in stage III group, median PFS after IRE was higher in IRE-NK (9.3 months) than in IRE (8.1 months, *P* = 0.0465, Figure 4A), median OS after IRE was higher in IRE-NK (13.2 months) than in IRE (11.4 months, *P* = 0.0411, Figure 4B), and median PFS after IRE who received multiple NK was higher than who just received single NK (9.8 months vs.8.1 months, *P* = 0.0423, respectively, Figure 5A), similarly, median OS after IRE who received multiple NK was higher than who received single NK (13.9 months vs.12.3 months, *P* = 0.0524, respectively, Figure 5B); in stage IV group, there was no difference of median PFS between IRE and IRE-NK (5.0 months vs.5.3 months, *P* > 0.05, respectively, Figure 4C), but median OS was higher in IRE-NK (9.8 months) than in IRE (8.7 months, *P* = 0.0397, Figure 4D), and median PFS and OS who received multiple NK were a little more than just received single NK, but they were both no difference (both *P* > 0.05, Figure 5C, 5D).

## DISCUSSION

For the majority of patients with advanced PC, tumor is not suitable to resect. The best options for metastatic patients are chemotherapy, radiofrequency ablation, and other palliative therapies, but the median survival time was only 6–11 months [35, 36]. IRE is a feasible and safe technique for PC ablation and [37, 38] has the potential to preserve the adjacent tissues, such as the nerves, vessels, and bile duct. Fuda Cancer Hospital (Guangzhou, China) has been conducting a prospective study of percutaneous IRE ablation of local advanced pancreatic cancer (LAPC) earliest by Dr. Niu which also validated its safety and efficacy [39].

In previous studies, the researchers were limited to IRE ablation of pancreatic cancer with stage III [37, 40, 41], Martin RG, et al propose that as long as the patient has not developed metastatic disease and the maximum axial diameter is not above 4.0 cm after induction therapy, then we would recommend proceeding with IRE therapy. However, in our study, we performed IRE in patients with stage III/IV PC, and the mean diameter of tumor > 4 cm, CT revealed no injury to the pancreatic duct, bile duct, or surrounding intestinal lumen post-treatment. The main results of our study indicate that IRE treatment has clinical significance for > 5cm tumors for patients with stage III pancreatic cancer and for patients with stage IV pancreatic cancer within the criteria for a limited metastatic tumor. This finding is in different to previously published inclusion criteria. Scheffer HJ, et al. [41] retrospectively examined the efficacy of IRE for locally advanced pancreatic cancer (stage III) and reported PFS and OS was 8 months and 11 months, respectively, in our study, the mean tumor diameter of 5.01 ± 1.06 cm patients with stage III pancreatic cancer who had a better median OS (12.2 months) and the same PFS (8.1 months). But there was no exactly report about how IRE effected the median PFS and OS of stage IV PC so far, we exhibited them firstly (5.0 months and 9.1 months, respectively). However, due to the vast majority of advanced PC patients have been confirmed the existence of the distal metastasis, IRE only treat primary tumors or metastases, possible role limited for advanced PC cell metastasis.

It is increasingly clear that cancer occurence and development in HCC patients are affected by tumor immune [42, 43], indicating that immune-based therapy could be an effective treatment option for patients with LAPC. Our previous research of Cryo-DC/CIK treatment with in metastatic PC [44] and HCC [45] has displayed a good clinical outcome. Suffered from cryotherapy, vast antigen released continuously, which stimulated the immune system, but CIK could not kill the tumor cell directly, so we brought in NK cells by understanding NK function. In our study, we attempted to investigate the safety and clinical efficacy of percutaneous IRE combined with allogeneic natural killer cell therapy for treating unresectable PC, then the median PFS, OS, RR and DCR were assessed. To our surprise was that allogeneic NK cells combined with IRE for unresectable PC exhibited a synergistic effect, significantly enhanced the immune function of patients (Table 4), the increasing number of NK cells after allogeneic NK cells immunotherapy may be related to ectogenic NK cells amplified *in vivo*, because NK cells were infused in the period of logarithmic phase which owned the best activity and amplified sequentially, hence, the patients who received NK therapy could increase the number of NK cells *in vivo*, and enhancing immune function. Most of all the median PFS and OS of stage III group were both higher in IRE-NK than who just received IRE, and multiple NK infusion showed a better median PFS and OS than single NK; the same to stage IV, the median OS was higher in IRE-NK than who underwent only IRE, but multiple NK infusion was no effect to median PFS and OS. And we found that the patients who underwent IRE-NK had a better RR in stage III and higher DCR in stage IV. Facts proved that this comprehensive therapy was also safety and efficacy.

For decades, natural killer (NK) NK cells existed as “non-specific” killer cells were different from CTL or other immunocytes identified the target. We have learned that NK cells are trained to recognize “non-self” histocompatibility antigens (human leukocyte antigen, HLA) on the surface of cells through their killer cell immunoglobulin-like (KIR) receptors. Recent discoveries that better explain how NK cells recognize and kill their targets and their ability to produce immune-active cytokines have made them more attractive tools for immunotherapy. In view of this, we brought NK cells into this clinical research. In our previous report [46], showed a good outcome for allogenic NK cell immunotherapy to advanced renal cell cancer Therefore, we used allogeneic NK cell adoptive therapy in this study. Facts proved that it was tolerant and efficacy. But whether the current number and purity of NK cells would be the optimal dose or not which is worthy us considering, maybe, we will carry out the dose grope in the future.

In conclusion, in this single-center, prospective study, we provided evidence that allogeneic NK cell therapy combined with percutaneous IRE has better median PFS and OS for patients with unresectable PC, which provides a potential therapeutic pattern.

## MATERIALS AND METHODS

### Ethics

This clinical research was approved by the Guangzhou Fuda Cancer Hospital ethics committee. In accordance with the Declaration of Helsinki, written informed consent was obtained from each participant.

### Patients

**Figure 6:**
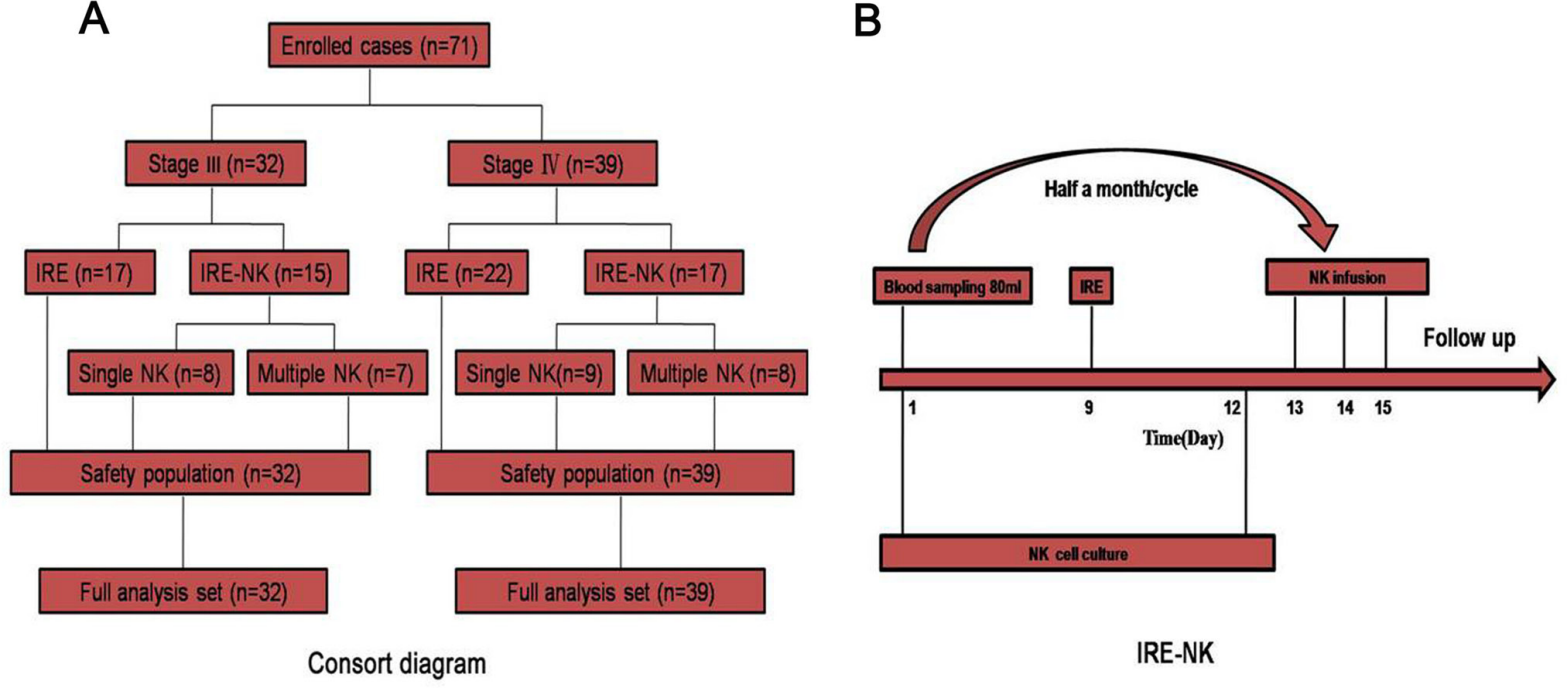
(**A**) Consort diagram. The patients were divided into stage III (32 patients, 17 patients received only IRE and 15 patients received IRE-NK: 8 patients underwent a course of NK and 7 patients underwent ≥ 3 courses) and stage IV (39 patients, 22 patients received only IRE and 17 patients received IRE-NK: 9 patients underwent a course of NK and 8 patients underwent ≥ 3 courses); (**B**) Procedure of IRE-NK therapy. All enrolled patient's kinsfolk were informed and peripheral blood collected for NK at 7days before IRE, IRE carried out at day 9, and at day 12, NK cell completed culture and infused intravenously at d 13~15.

This was a prospective study of the therapeutic effects of the combined treatment for patients with unresectable (Stage III/IV) pancreatic cancer enrolled between July 2016 and May 2017. We enrolled 71 patients using the following criteria: (1) expected survival > 3 months; (2) age 30–80 years; (3) Karnofsky performance status (KPS) > 60; (4) platelets ≥ 80 × 10^9^/L, white blood cells ≥ 3 × 10^9^/L, neutrophils ≥ 2 × 10^9^/L, hemoglobin ≥ 90 g/L, prothrombin time–international normalized ratio (0.8–1.5), adequate hepatic function (bilirubin < 20 μM, aminotransferase < 60 U/L) and renal function (serum creatinine < 130 μM, serum urea < 10 mM); (5) APC confirmed by pathology and/or imaging; (6) stage IV patients were limited to metastatic tumors <4 and the maximum diameter of metastatic tumors < 4 cm; (7) absence of level 3 hypertension, severe coronary disease, myelosuppression, respiratory disease, acute or chronic infection, and autoimmune diseases. The patients were divided into stage III (32 patients, 17 patients received only IRE and 15 patients received IRE-NK: 8 patients underwent a course of NK and 7 patients underwent ≥ 3 courses ) and stage IV (39 patients, 22 patients received only IRE and 17 patients received IRE-NK: 9 patients underwent a course of NK and 8 patients underwent ≥ 3 courses, Figure 6A).

### IRE procedure

All patients underwent neuromuscular blockade and general anesthesia. Computed tomography (CT) and ultrasound were used to guide electrode insertion, and IRE was synchronized to deliver electrical pulses coordinating with the cardiac rhythm. The distance between the electrodes was 1.5–2.0 cm. One or more pullbacks were performed if the target region was > 2 cm in diameter. After the ablation, the patient was transferred to the intensive care unit for overnight observation, and then transferred to the general ward after no acute complications were confirmed. Relevant treatment was administered if there were any complications. Two surgeons (L.Z.N. and L.Z.) with 4–8 years of experience in image-guided tumor ablation performed all procedures.

### CT examination

The patients were required to undergo plain CT and enhanced CT at 1 week pre-treatment, and followed at 1 month and 2 or 3 months post-treatment. The maximum diameter was measured and compared pre-treatment and post-treatment.

### NK cell therapy

For NK cells culture, after isolated PBMC from whole blood, using the Human HANK Cell *In Vitro* Preparation Kit (Hank Bioengineering Co., Ltd, Shenzhen, China), including the lethally radiated K562-mb15–41BBL (K562D2) stimulatory cells [47], plasma treatment fluid, lymphocyte culture fluid additives, serum-free medium additives and cell infusion additives. It is dedicated for the expansion and activation of NK cells in peripheral blood or umbilical cord blood mononuclear cells *in vitro*, the preparation of NK cells with higher quantity, purity and activity, namely HANK cells [48]. The final cell count and quality control inspection were performed at day 9 of culture, and the qualified indicators included proportion of living cells ≥ 90%, proportion of CD56 + CD3- cells ≥ 85% (detection by flowcytometry was shown previously [48]), endotoxin content ≤ 1 EU/ml, cell viability ≥ 80% (K562 cells were used as target cells, cytotoxicity assay was shown previously [48]), Bacteria, fungi and mycoplasma culture negative.

80 ml peripheral blood from allogenic donors was drawn 7 days before IRE and the immunotherapy was given 3 days after IRE. Approximately 8–10 billion HANK cells may be harvested after culture from 80 ml of peripheral blood. After 12 days of cell culture, the NK cells were divided into three groups and intravenously infused into the patients from Day 13 to 15. All cell preparation processes were performed by the same technician and assessed by another technician. Each patient must two cycles NK therapy continuously as a course.

For donor selection, the killer cell immunoglobulin-like receptors (KIRs) genotyping should be mismatched to the human leukocyte antigen (HLA) class I molecules of the patient [48–52]. We used PCR-SSP to detect the KIR/HLA-Cw which can get the result on the day.

### IRE-NK therapy

At 7 days before IRE, the relatives of the enrolled patients were informed and their peripheral blood was collected to obtain the NK cells. IRE was carried out on day 9, and on day 12, the NK cell culture was completed, and the cells were infused intravenously on day 13–15 (Figure 6B). Each patient was required to accept two continuous cycles as a course of NK after IRE.

### Safety evaluation index

Adverse events and complications: Adverse events during treatment and post-treatment, and immediate post-treatment complications, were closely observed and recorded.

The patient serum amylase index was checked pre-IRE and on day 1, 3, and 7 post-IRE to determine whether there was pancreatitis.

### Curative effect evaluation index

Detection of immune function: 2 mL peripheral blood was drawn to detect immune function and was assessed using flow cytometry (FACSCanto™ II; BD, Grand Island, NY, USA). The tested indices included lymphocyte number and function in the patients’ peripheral blood. BD multitest 6-color TBNK reagent (no. 644611) was used to detect the number of CD3^+^CD4^+^ cells (95% range: 441–2156/μL), CD3^+^CD8^+^ cells (95% range: 125–1312/μL), total CD3^+^ cells (95% range: 603–2990/μL), CD3^-^CD19^+^ cells (95% range: 107–698/μL), and CD3^-^CD16^+^CD56^+^ cells (95% range: 95–640/μL). BD Cytometric Bead Array Human Th1/Th2 Cytokine Kit II (no. 551809) was used to detect the expression levels of interleukin-2 (IL-2; 95% range: 8–12.5 pg/mL), IL-4 (95% range: 3.5–6 pg/mL), IL-6 (95% range: 2.7–8.5 pg/mL), IL-10 (95% range: 1.8–4 pg/mL), tumor necrosis factor (TNF; 95% range: 1.7–2.5 pg/mL), and interferon-γ (IFN-γ; 95% range: 1.5–4 pg/mL). The tests were performed according to the protocols in the instruction manuals. Results above or within the reference range were defined as normal immune function; one or more results below the reference range were defined as immune dysfunction. Peripheral blood drawn was 1 day before IRE and 3 days after IRE or IRE-NK.

Carbohydrate antigen 19–9 (CA19–9) expression was tested pre-treatment and at day 1, 7, and 1 month post-treatment.

Imaging changes: The World Health Organization published the tumor curative effect evaluation standard as a main study objective for observing tumor change [53]. Based on the degree of change of the largest transverse diameter, the therapeutic effect of the two treatments was divided into complete response (CR), arterial enhancement imaging of all target lesions disappeared; partial response (PR), the diameter sum reduction of target lesions was > 30%; stable disease (SD), tumor regression failed to achieve PR or tumor progression did not develop progressive disease (PD); and PD, the diameter sum progression of tumor was > 20%. To accurately observe the effect, we compared the sum area of all tumors before and after treatment. The recent curative effect must have been maintained at > 4 weeks; CR + PR denoted the effective rate (RR).

Follow-up: The patients were required to undergo plain CT and enhanced CT at 1 week pre-treatment, and followed at 1 month and 2 months post-treatment. The endpoints of interest were progression-free survival (PFS) and overall survival (OS). PFS was defined as the interval between IRE and local relapse, distant metastasis, or death, whichever occurred first. OS was calculated as the interval between the date of IRE and the date of death from any cause. All patients after treatment are focused by our manual and intelligent follow-up system persistently.

### Evaluation and statistical analysis

Complications were recorded and classified in accordance with the Common Terminology Criteria of Adverse Events v4.0 [54]. Radiographic local tumor control was assessed using image-guided tumor ablation criteria [55]. The basic characteristics of the two groups were compared using the chi-square test; immunity detection result data are presented as the mean ± standard deviation; the changes of imaging were compared using the Student's *t*-test; local and systemic adverse events were marked in the nursing records; the median PFS and OS was analyzed by Kaplan–Meier statistical method and using the log-rank test to compare. Significant differences were indicated by *P* < 0.05, *P* < 0.01 or *P* < 0.001. All analyses were conducted using GraphPad software (GraphPad, San Diego, CA, USA).

## Abbreviations

PC: pancreatic cancer
NK: natural killer
PFS: progression-free survival
OS: overall survival
RR: response rate
DCR: disease control rate
MHC: major histo-compatibility complex
KIRs: killer cell immunoglobulin-like receptors

## References

[1] Long J, Luo GP, Xiao ZW, Liu ZQ, Guo M, Liu L, Liu C, Xu J, Gao YT, Zheng Y, Wu C, Ni QX, Li M, Yu X (2014). Cancer statistics: current diagnosis and treatment of pancreatic cancer in Shanghai, China. Cancer Lett.

[2] Pisters KM (2005). Adjuvant chemotherapy for non-small-cell lung cancer—the smoke clears. N Engl J Med.

[3] Keller SM, Adak S, Wagner H, Herskovic A, Komaki R, Brooks BJ, Perry MC, Livingston RB, Johnson DH, Eastern cooperative oncology group (2000). A randomized trial of postoperative adjuvant therapy in patients with completely resected stage ii or iiia non-small-cell lung cancer. N Engl J Med.

[4] Felip E, Martinez-Marti A, Martinez P, Cedres S, Navarro A (2013). Adjuvant treatment of resected nonsmall cell lung cancer: state of the art and new potential developments. Curr Opin Oncol.

[5] Vulfovich M, Rocha-Lima C (2008). Novel advances in pancreatic cancer treatment. Expert Rev Anticancer Ther.

[6] Rombouts SJ, Vogel JA, van Santvoort HC, van Lienden KP, van Hillegersberg R, Busch OR, Besselink MG, Molenaar IQ (2015). Systematic review of innovative ablative therapies for the treatment of locally advanced pancreatic cancer. Br J Surg.

[7] Keane MG, Bramis K, Pereira SP, Fusai GK (2014). Systematic review of novel ablative methods in locally advanced pancreatic cancer. World J Gastroenterol.

[8] Silk M, Tahour D, Srimathveeravalli G, Solomon SB, Thornton RH (2014). The state of irreversible electroporation in interventional oncology. Semin Intervent Radiol.

[9] Kluger MD, Epelboym I, Schrope BA, Mahendraraj K, Hecht EM, Susman J, Weintraub JL, Chabot JA (2016). Single-institution experience with irreversible electroporation for t4 pancreatic cancer: First 50 patients. Ann Surg Oncol.

[10] Martin RC, Kwon D, Chalikonda S, Sellers M, Kotz E, Scoggins C, McMasters KM, Watkins K (2015). Treatment of 200 locally advanced (stage iii) pancreatic adenocarcinoma patients with irreversible electroporation: Safety and efficacy. Annals of Surgery.

[11] Paiella S, Butturini G, Frigerio I, Salvia R, Armatura G, Bacchion M, Fontana M, D’Onofrio M, Martone E, Bassi C (2015). Safety and feasibility of Irreversible Electroporation (IRE) in patients with locally advanced pancreatic cancer: results of a prospective study. Dig Surg.

[12] Narayanan G, Hosein PJ, Arora G, Barbery KJ, Froud T, Livingstone AS, Franceschi D, Rocha Lima CM, Yrizarry J (2012). Percutaneous irreversible electroporation for downstaging and control of unresectable pancreatic adenocarcinoma. J Vasc Interv Radiol.

[13] Aerts JG, Hegmans JP (2013). Tumor-specific cytotoxic T cells are crucial for efficacy of immunomodulatory antibodies in patients with lung cancer. Cancer Res.

[14] Lv M, Xu Y, Tang R, Ren J, Shen S, Chen Y, Liu B, Hou Y, Wang T (2014). miR141-CXCL1-CXCR2 signaling-induced Treg recruitment regulates metastases and survival of non-small cell lung cancer. Mol Cancer Ther.

[15] Topalian SL, Hodi FS, Brahmer JR, Gettinger SN, Smith DC, McDermott DF, Powderly JD, Carvajal RD, Sosman JA, Atkins MB, Leming PD, Spigel DR, Antonia SJ (2012). Safety, activity, and immune correlates of anti-PD-1 antibody in cancer. N Engl J Med.

[16] Thomas A, Hassan R (2012). Immunotherapies for non-small-cell lung cancer and mesothelioma. Lancet Oncol.

[17] Hasegawa T, Suzuki H, Yamaura T, Muto S, Okabe N, Osugi J, Hoshino M, Higuchi M, Ise K, Gotoh M (2014). Prognostic value of peripheral and local forkhead box P3(+) regulatory T cells in patients with non-small-cell lung cancer. Mol Clin Oncol.

[18] Hanahan D, Weinberg RA (2011). Hallmarks of cancer: the next generation. Cell.

[19] Spellman A, Tang SC (2016). Immunotherapy for breast cancer: past, present, and future. Cancer Metastasis Rev.

[20] Hadden JW (1999). The immunology and immunotherapy of breast cancer: an update. Int J Immunopharmacol.

[21] Kubo M, Morisaki T, Kuroki H, Tasaki A, Yamanaka N, Matsumoto K, Nakamura K, Onishi H, Baba E, Katano M (2003). Combination of adoptive immunotherapy with Herceptin for patients with HER2-expressing breast cancer. Anticancer Res.

[22] Drake CG, Jaffee E, Pardoll DM (2006). Mechanisms of immune evasion by tumors. Adv Immunol.

[23] Zhao Y, Hu J, Li R, Song J, Kang Y, Liu S, Zhang D (2015). Enhanced NK cell adoptive antitumor effects against breast cancer *in vitro* via blockade of the transforming growth factor-β signaling pathway. Onco Targets Ther.

[24] Cheng M, Chen Y, Xiao W, Sun R, Tian Z (2013). NK cell-based immunotherapy for malignant diseases. Cell Mol Immunol.

[25] Wang D, Zhang B, Gao H, Ding G, Wu Q, Zhang J, Liao L, Chen H (2014). Clinical research of genetically modified dendritic cells in combination with cytokine-induced killer cell treatment in advanced renal cancer. BMC Cancer.

[26] Li JJ, Gu MF, Pan K, Liu LZ, Zhang H, Shen WX, Xia JC (2012). Autologous cytokine-induced killer cell transfusion in combination with gemcitabine plus cisplatin regimen chemotherapy for metastatic nasopharyngeal carcinoma. J Immunother.

[27] Pan K, Guan XX, Li YQ, Zhao JJ, Li JJ, Qiu HJ, Weng DS, Wang QJ, Liu Q, Huang LX, He J, Chen SP, Ke ML (2014). Clinical activity of adjuvant cytokine-induced killer cell immunotherapy in patients with post-mastectomy triple-negative breast cancer. Clin Cancer Res.

[28] Pan K, Li YQ, Wang W, Xu L, Zhang YJ, Zheng HX, Zhao JJ, Qiu HJ, Weng DS, Li JJ, Wang QJ, Huang LX, He J (2013). The efficacy of cytokine-induced killer cell infusion as an adjuvant therapy for postoperative hepatocellular carcinoma patients. Ann Surg Oncol.

[29] Ljunggren HG, Malmberg KJ (2007). Prospects for the use of NK cells in immunotherapy of human cancer. Nat Rev Immunol.

[30] Yang L, Ren B, Li H, Yu J, Cao S, Hao X, Ren X (2013). Enhanced antitumor effects of DC-activated CIKs to chemotherapy treatment in a single cohort of advanced non-small-cell lung cancer patients. Cancer Immunol Immunother.

[31] Zhong R, Han B, Zhong H (2014). A prospective study of the efficacy of a combination of autologous dendritic cells, cytokine-induced killer cells, and chemotherapy in advanced non-small cell lung cancer patients. Tumour Biol.

[32] Li R, Wang C, Liu L, Du C, Cao S, Yu J, Wang SE, Hao X, Ren X, Li H (2012). Autologous cytokine-induced killer cell immunotherapy in lung cancer: a phase II clinical study. Cancer Immunol Immunother.

[33] Zhong R, Teng J, Han B, Zhong H (2011). Dendritic cells combining with cytokine-induced killer cells synergize chemotherapy in patients with late-stage non-small cell lung cancer. Cancer Immunol Immunother.

[34] Han RX, Liu X, Pan P, Jia YJ, Yu JC (2014). Effectiveness and safety of chemotherapy combined with dendritic cells co-cultured with cytokine-induced killer cells in the treatment of advanced non-small-cell lung cancer: a systematic review and meta-analysis. PLoS One.

[35] Eisenhauer EA, Therasse P, Bogaerts J, Schwartz LH, Sargent D, Ford R, Dancey J, Arbuck S, Gwyther S, Mooney M, Rubinstein L, Shankar L, Dodd L (2009). New response evaluation criteria in solid tumours: revised RECIST guideline (version 1.1). Eur J Cancer.

[36] Goldberg SN, Grassi CJ, Cardella JF, Charboneau JW, Dodd GD, Dupuy DE, Gervais D, Gillams AR, Kane RA, Lee FT, Livraghi T, McGahan J, Phillips DA, Society of Interventional Radiology Technology Assessment Committee, International Working Group on Image-Guided Tumor Ablation (2005). Image-guided tumor ablation: standardization of terminology and reporting criteria. Radiology.

[37] Martin RC (2013). Irreversible electroporation of locally advanced pancreatic head adenocarcinoma. J Gastrointest Surg.

[38] Martin RC, McFarland K, Ellis S, Velanovich V (2013). Irreversible electroporation in locally advanced pancreatic cancer: potential improved overall survival. Ann Surg Oncol.

[39] Zhang Y, Shi J, Zeng J, Alnagger M, Zhou L, Fang G, Long X, Pan Z, Li Y, Chen J, Xu K, Qian W, Niu L (2017). Percutaneous irreversible electroporation for ablation of locally advanced pancreatic cancer: experience from a chinese institution. Pancreas.

[40] Martin RC (2015). Irreversible electroporation of locally advanced pancreatic neck/body adenocarcinoma. J Gastrointest Oncol.

[41] Martin RC, Durham AN, Besselink MG, Iannitti D, Weiss MJ, Wolfgang CL, Huang KW (2016). Irreversible electroporation in locally advanced pancreatic cancer: A call for standardization of energy delivery. J Surg Oncol.

[42] Niu L, Xu K, Mu F (2012). Cryosurgery for lung cancer. J Thorac Dis.

[43] Izumi Y, Oyama T, Ikeda E, Kawamura M, Kobayashi K (2005). The acute effects of transthoracic cryoablation on normal lung evaluated in a porcine model. Ann Thorac Surg.

[44] Niu L, Chen J, He L, Liao M, Yuan Y, Zeng J, Li J, Zuo J, Xu K (2013). Combination treatment with comprehensive cryoablation and immunotherapy in metastatic pancreatic cancer. Pancreas.

[45] Niu LZ, Li JL, Zeng JY, Mu F, Liao MT, Yao F, Li L, Liu CY, Chen JB, Zuo JS, Xu KC (2013). Combination treatment with comprehensive cryoablation and immunotherapy in metastatic hepatocellular cancer. World J Gastroenterol.

[46] Lin M, Xu K, Liang S, Wang X, Liang Y, Zhang M, Chen J, Niu L (2017). Prospective study of percutaneous cryoablation combined with allogenic NK cell immunotherapy for advanced renal cell cancer. Immunol Lett.

[47] Imai C, Iwamoto S, Campana D (2005). Genetic modification of primary natural killer cells overcomes inhibitory signals and induces specific killing of leukemic cells. Blood.

[48] Zhang M, Daniel S, Huang Y, Chancey C, Huang Q, Lei YF, Grinev A, Mostowski H, Rios M, Dayton A (2010). Anti-West Nile virus activity of *in vitro* expanded human primary natural killer cells. BMC Immunol.

[49] Witt CS, Christiansen FT (2006). The relevance of natural killer cell human leucocyte antigen epitopes and killer cell immunoglobulin-like receptors in bone marrow transplantation. Vox Sang.

[50] Forte P, Baumann BC, Schneider MK, Seebach JD (2009). HLA-Cw4 expression on porcine endothelial cells reduces cytotoxicity and adhesion mediated by CD158a+ human NK cells. Xenotransplantation.

[51] Kunert K, Seiler M, Mashreghi MF, Klippert K, Schönemann C, Neumann K, Pratschke J, Reinke P, Volk HD, Kotsch K (2007). KIR/HLA ligand incompatibility in kidney transplantation. Transplantation.

[52] Moretta L, Moretta A (2004). Killer immunoglobulin-like receptors. Curr Opin Immunol.

[53] Miller AB, Hoogstraten B, Staquet M, Winkler A (1981). Reporting results of cancer treatment. Cancer.

[54] Chen AP, Setser A, Anadkat MJ, Cotliar J, Olsen EA, Garden BC, Lacouture ME (2012). Grading dermatologic adverse events of cancer treatments: the Common Terminology Criteria for Adverse Events Version 4.0. J Am Acad Dermatol.

[55] Goldberg SN, Grassi CJ, Cardella JF, Charboneau JW, Dodd GD, Dupuy DE, Gervais D, Gillams AR, Kane RA, Lee FT, Livraghi T, McGahan J, Phillips DA, Society of Interventional Radiology Technology Assessment Committee (2005). Image-guided tumor ablation: standardization of terminology and reporting criteria. J Vasc Interv Radiol.

